# High-Range and High-Linearity 2D Angle Measurement System for a Fast Steering Mirror

**DOI:** 10.3390/s23229192

**Published:** 2023-11-15

**Authors:** Boshi Du, Yong Lv, Lishuang Liu, Yang Liu

**Affiliations:** Instrumental Science and Optoelectronic Engineering Faculty, Beijing Information Science & Technology University, Beijing 100192, China; boshidu1997@gmail.com (B.D.); liulishuang@bistu.edu.cn (L.L.); 20141891@bistu.edu.cn (Y.L.)

**Keywords:** photoelectric sensors, fast steering mirror, large angle measurement system, diffuse reflection

## Abstract

In order to solve the problem of the insufficient range of the traditional fast mirror (FSM) angle measurement system in practical applications, a 2D large-angle FSM photoelectric angle measurement system based on the principle of diffuse reflection is proposed. A mathematical model of the angle measurement system is established by combining the physical properties of the diffuse reflecting plate, such as the rotation angle, rotation center, rotation radius, reflection coefficient and the radius of the diffuse reflecting surface. This paper proposes a method that optimizes the degree of nonlinearity based on this mathematical model. The system is designed and tested. The experimental results show that changing the diffuse reflection surface area can improve the nonlinearity of the angle measurement system effectively. When the radius of the diffuse reflection surface is 3.3 mm, the range is ±20°, the non-linearity is 0.74%, and the resolution can reach up to 2.3″. The system’s body is simple and compact. It is also capable of measuring a wider range of angles while linearity is guaranteed.

## 1. Introduction

Fast steering mirrors (FSMs) are often used to improve the performance of photoelectric imaging systems, such as airborne imaging systems and space-based optical surveillance systems [1,2,3]. It is necessary to measure the large deflection angle of the FSM to achieve a wider range of image acquisition capabilities. However, due to its compact spatial structure and fast response characteristics, the common large-angle angle measurement method and contact angle measurement method are not suitable for the structure of the FSM. Currently, common FSM angle measurement systems include capacitive sensor angle measurement systems, eddy current sensor angle measurement systems, digital encoder angle measurement systems, and photoelectric angle measurement systems. It is difficult for the above systems to achieve two-dimensional large-angle measurements of more than ±10°. Therefore, how to achieve a large-scale and high-precision angle measurement has become a research question for many scholars [4,5,6,7,8,9,10,11,12,13,14].

Yue Fan [15] demonstrated an angle measurement method based on an eddy current sensor which achieved an angle measurement accuracy of 1.1 arcsec in the range of ±1.5°. Lin D. [16] introduced a two-axis measurement system based on the principle of a capacitance sensor, with a measurement range of ±5°. OIM101 Inch [17] achieved a nonlinearity of 1% and a resolution of 1″ in the range of ±1.5°. Xin Wu [18] achieved a nonlinearity of 1% in the range of ±1.25°. YingXue Ni [19] achieved a measurement range of ±2° and a nonlinearity of 0.72% through specular reflection and QD sensors. ShaoKang Hung [20] established a spot center positional algorithm and realized a measurement of ±10° and a resolution of 0.0265° by collecting the spot position through QPD. Mingguang Shan [21] proposed a differential angle measurement system based on optical fiber devices which can achieve a complete linear measurement in the range of ±6.1°. Based on the above research, the current two-dimensional FSM angle measurement system can only achieve high resolution and high linearity in a small range, and it is difficult to achieve large angle measurements. Therefore, achieving large angle measurements while ensuring the measurements’ accuracy has become a problem.

In this paper, based on the principle of diffuse reflection, a two-dimensional large-angle FSM angle measurement system based on differential optical power was proposed. By analyzing the distribution of diffuse reflection light on the surfaces of different photodiode diffuse reflectors, a mathematical model was established to study the change in the differential optical power with the radius of the diffuse reflection surface. Then, an experimental model was built to test the static performance of the angle measurement system. Later, the correctness of the mathematical model was verified, a large angle measurement in the range of ±20° was realized, and the nonlinearity of the angle measurement system was optimized.

## 2. Deflection Angle Detection Principle

**Figure 1:**
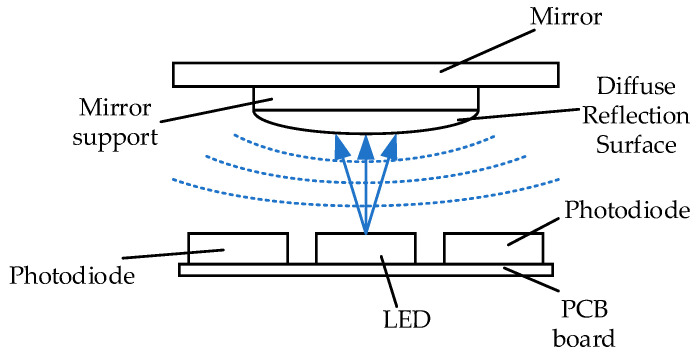
Optical structure design of 2D large angle measurement system.

**Figure 2:**
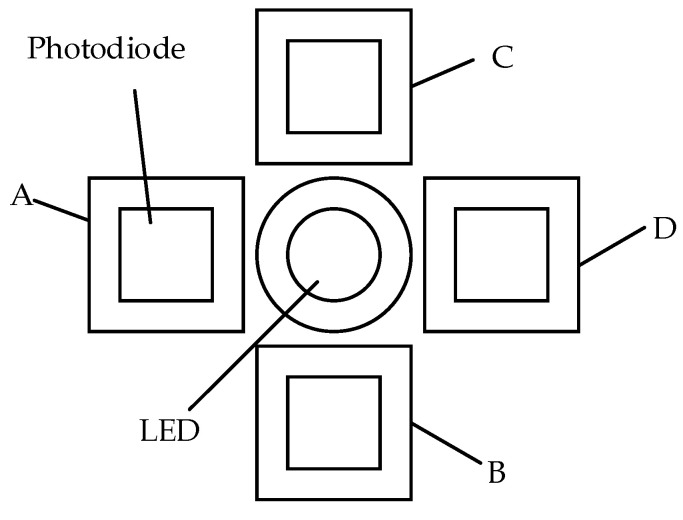
Structural design of optical signal transmitting and receiving Circuit.

Figure 1 shows the optical structure of the two-dimensional large-angle angle measurement system, which is divided into upper and lower parts. The upper part comprises the mirror, mirror support, and the diffuse reflection surface of the FSM. The lower part comprises the optical signal transmitting and receiving circuit, whose structure is shown in Figure 2. The acquisition circuit is fixed on a small PCB board. The LED light source in the middle directly faces the center of the diffuse reflector in a spatial position. Four photodiodes are evenly distributed around the light source and are divided into two groups, A and D and B and C. The two groups are placed orthogonally.

When the LED light source irradiates the diffuse reflector, the light is reflected through the diffuse reflection surface. Then, the light is reflected to the optical signal acquisition plate in an irradiation mode similar to the Lambert light source. After the photodiode on the surface of the optical signal acquisition plate is illuminated, different photocurrents are generated according to the change in the surface optical power. When the angle of the mirror of the FSM changes, the angle of the diffuse reflection surface will also change, which will affect the distribution of the reflected light intensity on the photoelectric acquisition plate so that the surface light power of the four photodiodes is different, and the change in the angle of the FSM will be converted into the output change in the photocurrent generated by the photodiode. For example, when the FSM mirror swings in the direction of photodiode A, the diffuse reflection surface will swing in the direction of photodiode D. The optical power of the reflected light of the diffuse reflection surface falling on photodiode D will become larger, the optical power falling on photodiodes A, B, and C will become smaller, and the optical power on the B and C surfaces will change similarly. The relationship between the angle value and the differential optical power can be obtained from the difference in the optical power on the surfaces of the two pairs of photodiodes.

## 3. Establishment of a Theoretical Model for the Angle Measurement System

### 3.1. Diffuse Reflection Theory

**Figure 3:**
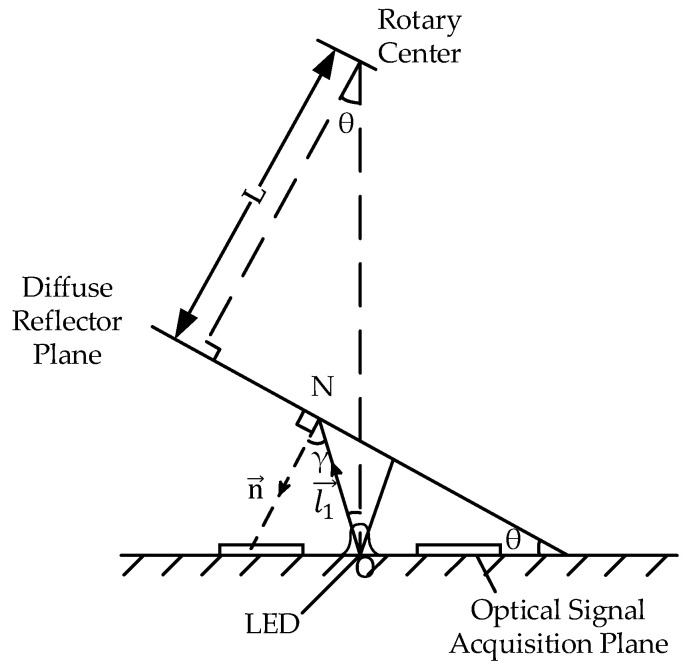
Plane diagram of the angle measurement system.

The angle measurement system realizes the test function by using the diffuse reflection theory. In the theoretical model, it is assumed that the diffuse reflector used in the experiment is an ideal diffuse reflector, namely, Lambert’s reflector, and its surface reflects light with equal intensity in all directions. According to the properties of Lambert’s reflector, when an arbitrary ray of light irradiates Lambert’s reflector, the intensity of the diffuse light is proportional to the cosine of the angle between the direction of the incident light and the normal direction of the incident point surface:

(1) $$I_{\mathrm{ldiff}}=k_{d}\;I_{l}\;\cos\theta$$

where k_d_ (0 < k_d_ < 1) represents the reflection coefficient of the material to the ambient light; Il represents the intensity of the point light source; θ represents the angle between the direction of the incident light and the normal of the vertex, which is called the incident angle (0° ≤ θ ≤ 90°); and I_ldiff_ represents the light intensity reflected by the interaction between the diffuse reflector and any light. Figure 3 is a plane map of the angle measurement system. In practice, in order to make the reflected light closer to the reflected light of an ideal Lambertian body, a material with a reflection coefficient close to 1 should be used, and the material used in this paper is polytetrafluoroethylene, with a reflection coefficient of 0.983.

Where θ represents the tilt angle of the diffuse reflection plane; γ represents the angle between the direction vector of the light source $\vec{l_{1}}$ and the normal vector of the diffuse reflection surface $\vec{n}$; and N point represents any irradiated point on the diffuse reflection surface. In this way, the light intensity reflected by any point on the diffuse reflection surface can be expressed as follows:

(2) $$I_{\mathrm{ldiff}}=k_{d}\;I_{l}\;\cos\gamma =k_{d}{\;I}_{l}\;\frac{\left|\vec{n}\;\cdot \;\vec{l_{1}}\right|}{\left|\vec{n}\right|\;\cdot \;\left|\vec{l_{1}}\right|}$$

**Figure 4:**
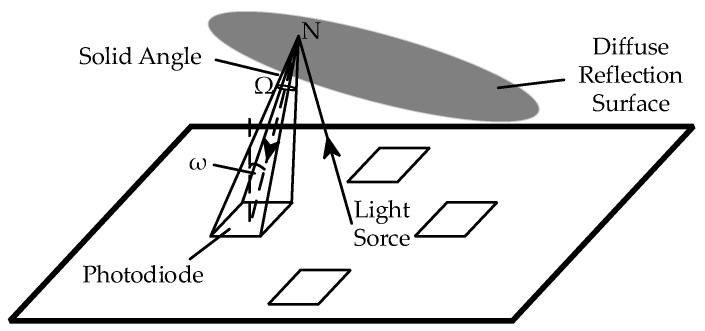
Diagram of a spatial solid angle.

Due to the diffuse reflection of the light, the reflection point N on the diffuse reflection surface can be considered a new point light source, which is a spherical wave with a light intensity of I_ldiff_, as shown in Figure 4.

The diffuse reflection surface reflects light to the surface of the photodiode, and the optical power per unit surface of the photodiode can be expressed as follows:

(3) dE=I dΩ

where dE represents the unit surface light power; I represents the light intensity; and dΩ represents the solid angle corresponding to the unit surface area, which can be expressed as

(4) $$d\Omega =\frac{\mathrm{dS}\;\cdot \;\cos\omega }{l^{2}}$$

where dS represents the unit surface area of the observation surface; ω represents the included angle between the normal of the observation surface and the incident light; and l represents the distance from the point light source to the center of the unit surface.

That is, after determining the solid angle Ω of the photodiode’s surface to the point light source N, the optical power of the light emitted by any point in the point light source on the photodiode’s surface can be acquired.

As shown in Figure 4, when integrating dΩ, ω changes. Since the surface area of the photodiode is very small, for any point N on the diffuse reflection surface, when integrating the diffuse reflection surface, the actual change range of ω is less than 7°. Therefore, ω can be simplified, that is, cosω in all unit area sources is considered approximately equal, and S can be defined as the surface area of the photodiode as follows:

(5) $$\Omega =\frac{S\;\cdot \;\cos\omega }{l^{2}}$$

Inserting Equations (2) and (4) into Equation (3), the surface optical power E of the photodiode reflected by any point on the diffuse reflection surface can be obtained.

(6) $$E=k_{d}\;I_{l}\;\frac{\left|\vec{n_{1}}\;\cdot \;\vec{l_{1}}\right|}{\left|\vec{n_{1}}\right|\;\cdot \;\left|\vec{l_{1}}\right|}\frac{S\;\cdot \;\cos\omega }{l^{2}}$$

### 3.2. Diffuse Reflection Model of Angle Measurement System

**Figure 5:**
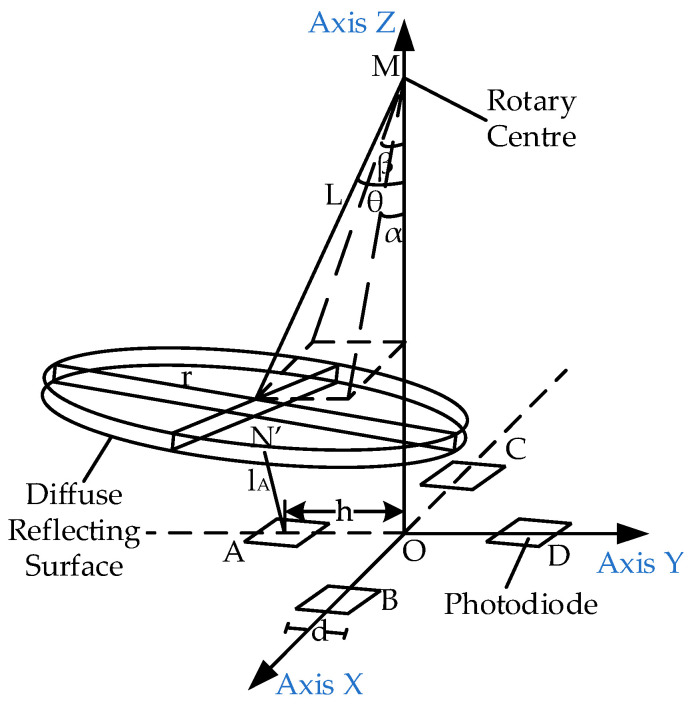
Space model of angle measurement system.

Based on the above theory, a spatial coordinate system is established with the light source as the origin. The light intensity IL of the light source is determined by the light distribution curve of the light source, and the light distribution curve is expressed as the function F (x, y, z). When the FSM mirror rotates, the diffuse reflection surface also rotates around the rotation center of the FSM mirror. The coordinates of the rotation center are M (0, 0, M), the radius of the diffuse reflection surface is L, and the diffuse reflection surface is a circular surface with a radius of r. The coordinates of the four photodiode centers are A (0, −h, 0), B (h, 0, 0), C (−h, 0, 0), and D (0, h, 0), and the photodiode is a square with a side length of d. The distances from any point N’ (x, y, z) on the diffuse reflection surface to the surfaces of the four photodiodes are l_A_, l_B_, l_C_, and l_D_, respectively, as shown in Figure 5.

Here, α represents the included angle between the turning radius and the xoz plane; β represents the included angle between the turning radius and the yoz plane; and θ represents the spatial angle of the turning radius, as follows:

(7) $$\tan\theta =\sqrt{\tan^{2}\alpha +\tan^{2}\beta }$$

The normal vector of the photodiode is the same as that of the xoy plane $\vec{n_{0}}=(0,\;0,\;1)$. The light vector of the light source to the diffuse reflection surface is defined as $\vec{n_{1}}=(x,\;y,\;z)$, the normal vector of the diffuse reflection surface is defined as $\vec{n_{2}}=(\tan\alpha ,\;\tan\beta ,\;-1)$, and the light vector of any point N′ on the diffuse reflection surface to the center point of photodiode A is defined as $\vec{n_{3}}=(x,\;y-\;h,\;z)$.

The vector in Equation (2) is extended from a plane vector to a space vector to obtain the diffuse reflection intensity of any point N′ (x, y, z) on the diffuse reflection surface, as follows:

(8) $$I_{\mathrm{ldiff}}^{\prime }=k_{d}I_{l}\frac{\left|-\vec{n_{1}}\;\cdot \;\vec{n_{2}}\right|}{\left|\vec{n_{1}}\right|\;\cdot \;\left|\vec{n_{2}}\right|}=k_{d\;}F\left(x,\;y,\;z\right)\frac{\mathrm{xtan}\alpha +\mathrm{ytan}\beta \;-\;z}{\sec\theta \;\cdot \;\sqrt{x^{2}+y^{2}+z^{2}}}$$

The distance between any point on the diffuse reflection surface and the center point of photodiode A is

(9) $$l_{A}=\sqrt{x^{2}+{(y\;-\;h)}^{2}+z^{2}}$$

where cosω can be expressed as

(10) $$\cos\omega =\frac{\left|-\vec{n_{3}}\;\cdot \;\vec{n_{0}}\right|}{\left|\vec{n_{3}}\right|\;\cdot \;\left|\vec{n_{0}}\right|}=\frac{z}{\sqrt{x^{2}+{(y\;-\;h)}^{2}+z^{2}}}$$

Inserting Equations (8)–(10) into Equation (6), the optical power from any point on the diffuse flat plate reflector to the surface of photodiode A can be obtained as follows:

(11) $${\text{∆}E}_{A}=\frac{k_{d}\;F\left(x,\;y,\;z\right)}{\sec\theta }\frac{\mathrm{xtan}\alpha +\mathrm{ytan}\beta -\;z}{\sqrt{x^{2}+y^{2}+{\;z}^{2}}}\frac{d^{2}\;\cdot \;z}{{\sqrt{x^{2}+{(y\;-\;h)}^{2}+z^{2}}}^{3}}$$

By integrating (11) on the diffuse reflection surface, the optical power of all diffuse reflection rays on the surface of the photodiode can be obtained. The method of obtaining the integral interval is as follows.

When the diffuse reflector rotates around the center of rotation, the space equation of the diffuse reflector plane is

(12) −xtanα − ytanβ+z+Lsecθ − M=0

The circular diffuse reflection surface can be expressed as a plane formed by the intersection lines between the diffuse reflection surface plane and the spatial cylindrical surface. The equation of the spatial cylindrical surface is

(13) $${\left(-\mathrm{xtan}\beta +\mathrm{ytan}\alpha \right)}^{2}+{\left[x+\tan\alpha \left(z\;-\;M\right)\right]}^{2}+{\left[y+\tan\beta \left(z-\;M\right)\right]}^{2}=r^{2}\sec^{2}\theta$$

Equation (14) of the projection surface P of the space plane on the xoy plane is obtained by combining function (12) with function (13), and the projection surface P is the integral interval.

(14) $$P\text{:}\left\{\begin{array}{c} x^{2}+y^{2}+{\left[-\mathrm{xtan}\alpha \;-\mathrm{ytan}\beta +\mathrm{Lsec}\theta \right]}^{2}\text{<}r^{2}+L^{2}\\ z=0 \end{array}\right.$$

By integrating optical power on the interval P, the optical power value of all diffuse reflection rays reflected on the surface of photodiode A can be obtained.

(15) $$E_{A}=k_{d}\;d^{2}\;(M\;-\;\mathrm{Lsec}\theta )\iint_{P}^{}\frac{\;F\left(x,\;y,\;z\right)}{\sqrt{x^{2}+y^{2}+z^{2}}}\frac{z}{{\sqrt{x^{2}+{(y\;-\;h)}^{2}+z^{2}}}^{3}}\mathrm{dxdy}$$

**Figure 6:**
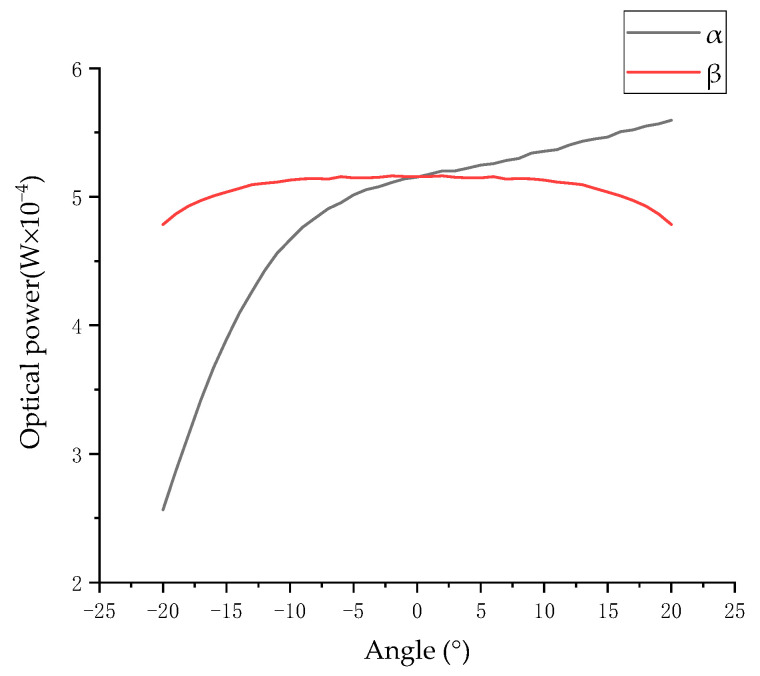
Surface optical power of a single photodiode.

Supposing that the light distribution curve L obeys second-order normal distribution; then, the light source opening angle is 17°, r = 5.0, L = 7.0, M = 10.0, d = 1.4, D = 3.5, k_d_ = 0.983, β = 0, and θ = α. When α changes from −20° to 20°, the changes in the surface light intensity of a single photodiode in the α and β directions are shown in Figure 6.

Figure 6 shows changes in the light intensity on the surface of a single photodiode. In principle, the angle can be measured by using a photodiode in both directions. However, as shown in Figure 6, within a large angle range, the nonlinearity of the angle measured by a single sensor in the α direction is 38.32%, and it is non-monotonic. For a non-monotonic system with poor linearity, the linearity can be optimized by difference. Therefore, the difference model is obtained by subtracting the optical power of the surfaces of the photodiodes A and D:

(16) $$E_{A-D}=k_{d}(M\;-\;\mathrm{Lsec}\theta )d^{2}\iint_{\pi }^{}\frac{\;F\left(x,\;y,\;z\right)}{\sqrt{x^{2}+y^{2}+z^{2}}}(\frac{z}{{\sqrt{x^{2}+{\left(y+h\right)}^{2}+z^{2}}}^{3}}-\frac{z}{{\sqrt{x^{2}+{(y\;-h)}^{2}+z^{2}}}^{3}})\mathrm{dxdy}$$

For the same reason, the difference model is obtained by subtracting the optical power of the surfaces of the photodiodes B and C:

(17) $$E_{B-C}=k_{d}(M\;-\;\mathrm{Lsec}\theta )d^{2}\iint_{P}^{}\frac{\;F\left(x,\;y,\;z\right)}{\sqrt{x^{2}+y^{2}+z^{2}}}(\frac{z}{{\sqrt{{\left(x\;-\;h\right)}^{2}+y^{2}+z^{2}}}^{3}}-\frac{z}{{\sqrt{{\left(x+h\right)}^{2}+y^{2}+z^{2}}}^{3}})\mathrm{dxdy}$$

**Figure 7:**
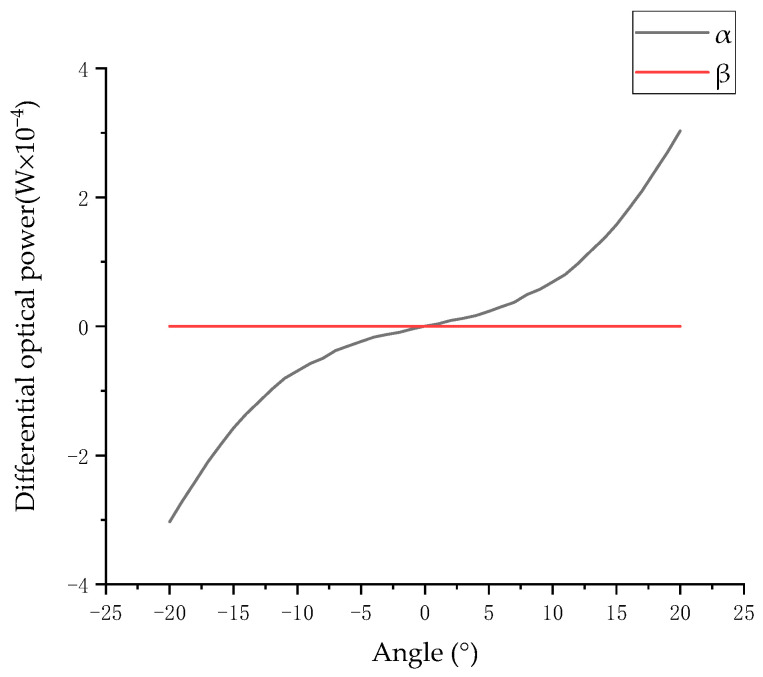
Relationship between the surface differential optical power and the angle of two pairs of photodiodes.

Putting r = 5.0, L = 7.0, M = 10.0, d = 1.4, D = 3.5, k_d_ = 0.983, β = 0, and θ = α[−20°, 20°] into (16) and (17), the differential optical power of the two groups of photodiode surfaces can be obtained, as shown in Figure 7.

In Figure 7, the nonlinearity of the differential optical power is 11.20%. It can be seen that when the optical power is used as the reference standard for the angle change, the differential form can effectively reduce the nonlinearity of the measurement results and improve the measurement accuracy.

**Figure 8:**
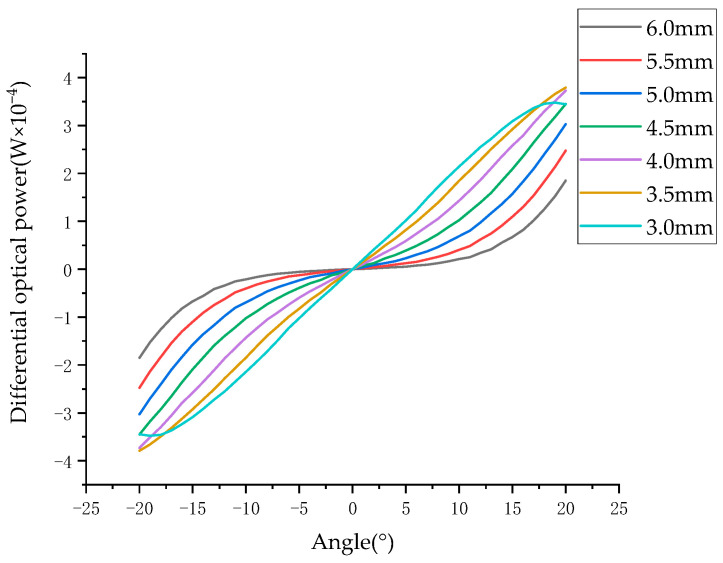
Relationship between the surface differential optical power and the angle at different radii.

In this way, the parameters in Equation (16) can be adjusted to reduce the nonlinearity. Among the five parameters r, L, M, d, and D, the radius of the diffuse reflection surface r most significantly influences the differential optical power ΔE. Keeping the other parameters unchanged, different r values are substituted into the equation to obtain the relationship between the light intensity difference and the radius of the diffuse reflection plate, as shown in Figure 8.

When the differential optical power changes with the angle, the greater the nonlinearity of the differential optical power, the greater the difference in the resolution of the angle measurement at different angles. As shown in Figure 8, when the radius of the diffuse reflection surface is 6 mm, the differential optical power does not obviously change within the angle range of −10°to 10°. As a result, the angle measurement system has poor angular resolution within the range of −10°to 10°. When the nonlinearity of the system is small, the system can clearly distinguish the changes in the differential splitting power with angle values between −20°and 20°.

Starting from the diffuse reflection surface radius of 6 mm, as the diffuse reflection surface radius decreases, the differential optical power changes approximately linearly. According to the above simulation data, compared with the other radii shown in Figure 8, when the diffuse reflection surface radius is 3.5, the minimum nonlinearity of the differential optical power is 1.8%. When the radius of the diffuse reflector is less than or equal to 3.0 mm, there is obvious non-monotonicity within the range of −20° to 20°, which makes a diffuse reflector with a radius of less than 3 mm unsuitable for angle measurement.

## 4. Experiments and Results

### 4.1. Experimental Structure of the Angle Measurement System

**Figure 9:**
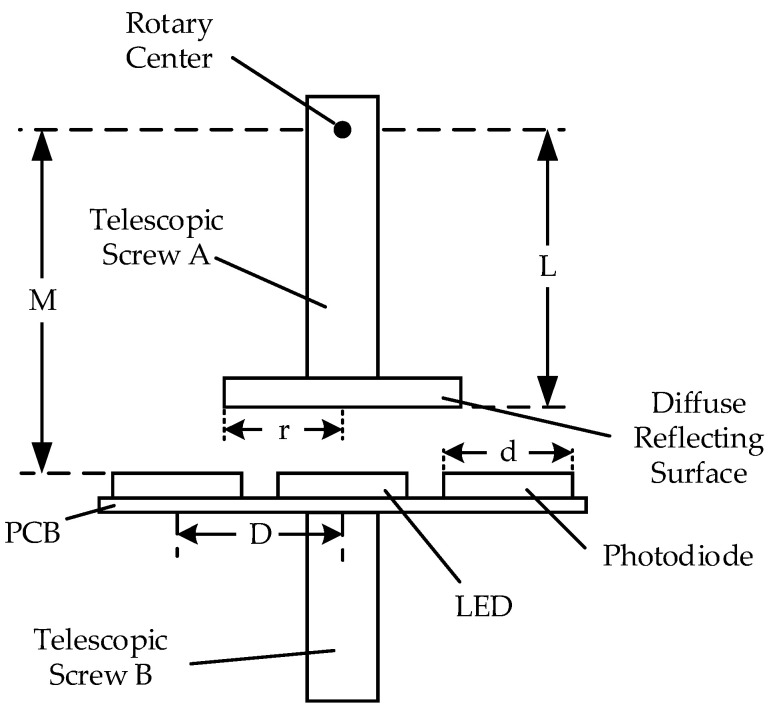
Schematic diagram of the testing apparatus structure.

To verify the validity of the model, a test mechanism was designed based on the principle of the simulation results, as shown in Figure 9.

The rotation radius L can be adjusted by adjusting the length of telescopic screw A, and the distance M between the LED light source and the rotation center can be adjusted by adjusting telescopic screw B. The LED and the photodiode are installed on the PCB plate according to the structure shown in Figure 2 to form an optical signal processing plate. The PCB is fixed on telescopic screw B, and the adjustment of the photodiode spacing D can be realized by replacing the PCB plate.

**Figure 10:**
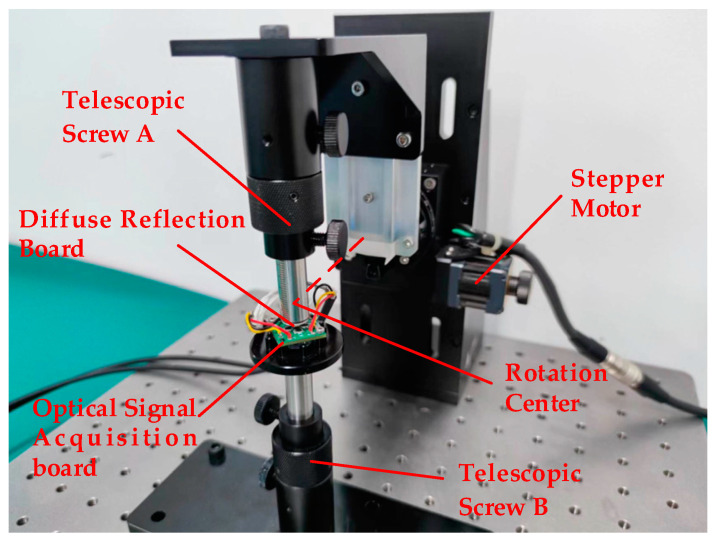
Static photoelectricity angle measurement system.

The test device was built according to the test structure. The light source of the test system adopted an SFH4441 LED with a wavelength of 940 nm, and the opening angle of the light source was 17°. For the material of the diffuse reflecting surface, the reflection coefficient of a 940 nm wavelength is 0.983. The model of the four photodiodes was PD15-22B/TR8, and the surface of each photodiode was a rectangular surface of 1.4 mm × 1.4 mm. Retractable screw A was fixed on the rotating platform through an adaptor, and the rotating platform was controlled by a stepper motor. The motor model was K431-60, with an accuracy of 0.004°. Figure 10 shows the test system.

When the rotating platform rotates, telescopic screw rod A rotates around the center of the rotating platform by following the adapter, driving the diffuse reflector to rotate around the rotating center. When the light source irradiates the diffuse reflector, the light intensity on the surfaces of the four photodiodes changes with the angle of the diffuse reflector, and the light intensity difference of the pair of photodiodes changes relatively. The differential voltage of the angle measuring circuit changes, and the analog circuit is converted into a digital signal via ADC, that is, the AD value corresponds to the light intensity difference. In this experiment, the deflection angle of the angle motion table is used as the theoretical value, and the AD value is the actual value. The AD value corresponds to the angle value, and the floating AD value corresponds to the measurement accuracy value. The relationship can be verified according to the corresponding relationship between the AD value and the angle.

### 4.2. Results and Analysis of the Deflection Angle Measurement System

**Figure 11:**
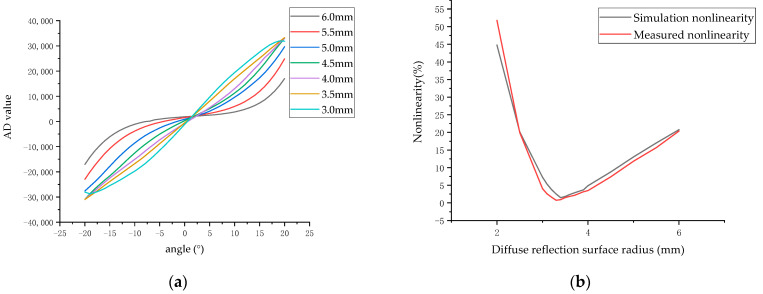
(**a**) The relationship between the angle value and the differential optical power for different diffuse surface radii; (**b**) the relationship between the diffuse reflection surface radius and the nonlinearity of the differential optical power.

The test system was set according to the following parameters. The distance from the center of the four photodiodes to the light source was 3.5 mm, the distance from the center of the diffuse reflection surface to the light source was 3.0 mm, and the distance from the diffuse reflection surface to the rotation center was 7.0 mm. The rotation angle of the diffuse reflection surface changed in the range of −20° to + 20°, and the radii of the diffuse reflection plate were 3.0 mm, 3.5 mm, 4.0 mm, 4.5 mm, 5.0 mm, 5.5 mm, and 6.0 mm, respectively. The rotation angle of the rotation center was adjusted by the electronically controlled rotating platform to obtain the experimental results, as shown in Figure 11.

As Figure 11a shows evidently, the measurement value changes as the angle value changes. When the radius of the diffuse reflection surface is less than 3.0 mm, the angle measurement system is non-monotonic within ±20°. When the radius of the diffuse reflection surface is more than 4.0 mm, the angle measurement system shows an evident nonlinearity trend. As the radius of the diffuse reflection surface increases, the variation in the differential optical power with the angle value increases. When the radius of the diffuse reflection surface is between 3.0 mm and 4.0 mm, there is a point of minimum nonlinearity. Figure 11b illustrates nonlinearity changes for different radii of the diffuse reflection surface. The simulated calculation results show that the system’s minimum nonlinearity is 0.81% when the radius of the diffuse reflection surface is 3.38 mm. The experimental tests were conducted around 3.38 mm. When the radius is 3.3 mm, nonlinearity is 0.74%; when the radius is 3.4 mm, the nonlinearity is 0.92%. Both Figure 8 and Figure 11a show that the experimental results are consistent with the simulation results.

In summary, the mathematical models (16) and (17) prove the correctness and feasibility of this angle measurement method based on the photodiodes’ differential optical power through simulated and experimental results. The experimental results are highly consistent with the simulation results. Therefore, this scheme is effective for the design of the angle measurement system of an FSM.

**Figure 12:**
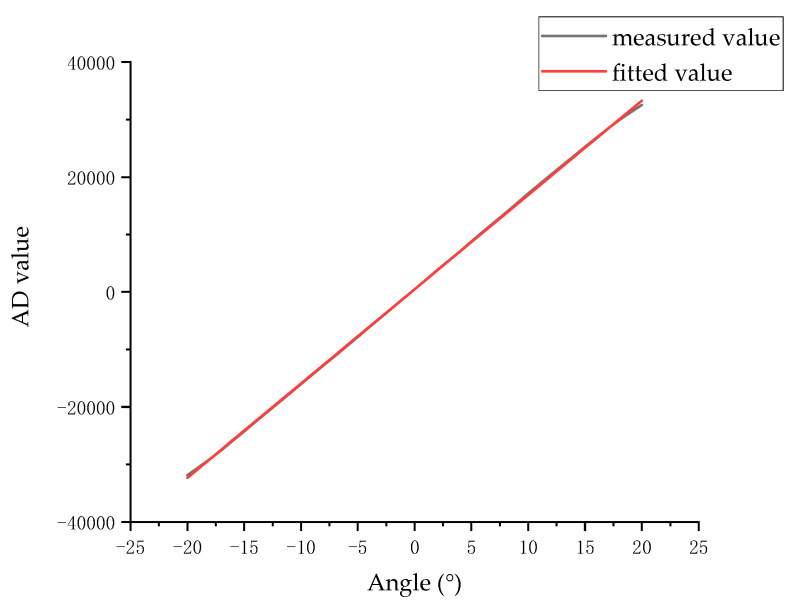
Measured values and fitting curves.

The diffuse reflection surface with a radius of 3.3 mm was analyzed. The AD value was tested in the range of −20° to 20°, and linear fitting was performed to obtain the measurement line and the fitting line, as shown in Figure 12.

The fitting function of the angle value and the differential AD value in the angle range of ±20° is

$$F\left(\theta \right)=1641\cdot \theta +482.7$$

The resolution of the angle measurement system is 2.3″. Combined with Figure 12, it can be seen that the fitting function is approximately coincident with the measured value, and the R2 value is 0.9999.

## 5. Conclusions

This paper investigates the working principle of diffuse reflection and analyzes the advantages and disadvantages of an angle measurement system for an FSM. It also proposes a solution for achieving large-angle measurement using diffuse reflection. A differential optical power angle measurement model which is applicable to various FSMs is established. The structural variables that actually affect the measurement results are clarified in the model. It also proposes an optimized method for nonlinearity in angle measurement. Through experiments and simulations, the validity of the model is proved. The model plays an auxiliary role in the structural design of an angle measurement system of a large-angle FSM. We also designed a testing apparatus to test the range, nonlinearity, and accuracy of the angle measurement system. The testing results demonstrate that this proposed system can achieve measurements within a ±20° angle range, a resolution of 2.3″, and an optimized nonlinearity of 0.74%. Compared to other 2D FSM angle measurement systems, it reaches larger measurement ranges, smaller nonlinearity, and is suitable for various FSM applications.

## Data Availability

No new data were created or analyzed in this study. Data sharing is not applicable to this article.
